# Comparative Metabolic Study of Two Contrasting Chinese Cabbage Genotypes under Mild and Severe Drought Stress

**DOI:** 10.3390/ijms23115947

**Published:** 2022-05-25

**Authors:** Lin Chen, Yongrui Shen, Wenjing Yang, Qiming Pan, Chao Li, Qingguo Sun, Qi Zeng, Baohua Li, Lugang Zhang

**Affiliations:** 1 State Key Laboratory of Crop Stress Biology for Arid Area, College of Horticulture, Northwest A&F University, Yangling 712100, China; chenlin1995@nwafu.edu.cn (L.C.); yangwenjing@nwafu.edu.cn (W.Y.); 2020055340@nwsuaf.edu.cn (Q.P.); lc1999@nwsuaf.edu.cn (C.L.); qguosun@nwafu.edu.cn (Q.S.); zengqi@nwafu.edu.cn (Q.Z.); 2 College of Life Science, Northwest A&F University, Yangling 712100, China; shenyongrui0926@nwafu.edu.cn

**Keywords:** Chinese cabbage, drought stress, metabolome, abscisic acid, glutathione

## Abstract

Chinese cabbage (*Brassica rapa* L. ssp. *pekinensis*) is an important leafy vegetable crop cultivated worldwide. Drought is one of the most important limiting factors for the growth, production and quality of Chinese cabbage due to its weak drought tolerance. In order to deepen the understanding of drought stress response in Chinese cabbage, metabolomics studies were conducted in drought−tolerant (DT) and drought−susceptible (DS) genotypes of Chinese cabbage under water deficit−simulated mild and severe drought stress conditions. A total of 777 metabolites were detected, wherein 90 of them were proposed as the drought−responsive metabolites in Chinese cabbage, with abscisic acid (ABA), serine, choline alfoscerate, and sphingosine as potential representative drought stress biomarkers. We also found that drought−tolerant and drought−susceptible genotypes showed differential metabolic accumulation patterns with contrasting drought response mechanisms. Notably, constitutively high levels of ABA and glutathione were detected in drought−tolerant genotype in all tested and control conditions. In addition, proline, sucrose, γ−aminobutyric acid, and glutathione were also found to be highly correlated to drought tolerance. This study is the first metabolomic study on how Chinese cabbage responds to drought stress, and could provide insights on how to develop and cultivate new drought−resistant varieties.

## 1. Introduction

Drought is one of the major environmental factors affecting agricultural production and food security, especially in arid and semi−arid areas where water supply is a major challenge [1,2]. Thus, developing new crop varieties with low water consumption is critical for sustaining agriculture and the environment [1].

Chinese cabbage, a fresh leafy vegetable with a high leaf water content and shallow root system, is widely cultivated and consumed around the world, especially in East Asia where the shortage of fresh water is a major challenge for agriculture. Therefore, it is of great importance to increase the drought tolerance of Chinese cabbage for its stable production. Genetic and molecular breeding has become increasingly important for improving drought tolerance of crops, including Chinese cabbage [3,4], and the understanding of the plant’s drought response would provide theoretical framework for breeding drought−resistant varieties.

Plants produce huge numbers of metabolites in order to sustain their growth and reproduction, as well as adapt to biotic and abiotic stresses. As plants are physically fixed and cannot move, their metabolic responses are key survival tools to deal with various environment stresses. In recent years, technological advances have been greatly developed and utilized to study plants in omics−based approaches, and metabolomics has become an efficient and important approach to gain panoramic views of how plants respond to stresses at the whole metabolism level [5,6]. However, there are only RNA−seq studies of Chinese cabbage’s responses to drought stress on the transcriptional level, while the metabolic responses at the whole metabolome level have not been reported yet [7,8,9].

A large number of metabolomics studies have been conducted to reveal drought stress responses in different plant species, and their metabolisms have been significantly affected by drought stress [10,11,12,13]. In Chinese cabbage, previous studies have shown that drought significantly influences the accumulation of multiple diverse groups of metabolites including glucosinolates, polyphenols, flavonoids, total antioxidant enzyme activities, catalases, and peroxidases in Chinese cabbage [9,14]. Therefore, we propose that Chinese cabbage may also systemically respond to drought stress at the metabolic level.

In this study, we characterized metabolic responses in two Chinese cabbage genotypes with contrasting drought tolerance under mild and severe drought by widely targeted metabolome technology. This study aimed to (i) identify drought−responsive metabolites and potential drought stress biomarkers in Chinese cabbage; (ii) compare the metabolic responses of Chinese cabbage genotypes with contrasting drought tolerance under mild and severe drought; and (iii) explore potential metabolites associated with increased drought tolerance and propose a metabolic response framework in Chinese cabbage under drought stress.

## 2. Results

### 2.1. Screening for Drought Tolerant and Susceptible Genotypes of Chinese Cabbage

The seed germination rate of 27 Chinese cabbage inbred lines was observed under 20% polyethylene glycol (PEG−6000) and two lines with extreme phenotypic variations were identified, with 14S837 having the highest germination rate (~80%) while 88S148 having no seed germination (Figure S1). These two lines were selected as candidates representing drought−tolerant (DT) and drought−susceptible (DS) genotypes, respectively.

**Figure 1:**
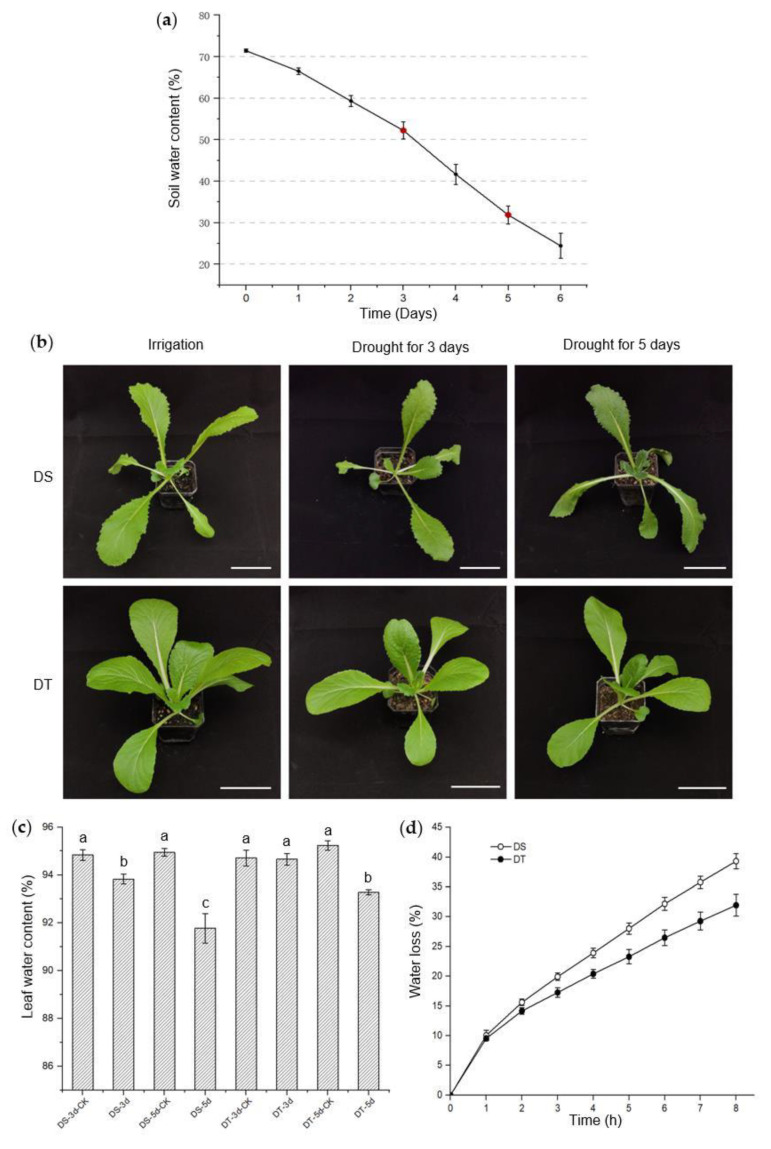
Phenotypic screening and investigations of drought−susceptible (DS) and drought−tolerant (DT) genotypes. (**a**) Soil water content during drought stress. Sampling time labeled in red; (**b**) Morphology of DS and DT after 3 and 5 days without watering. Scale bars, 7 cm; (**c**) Leaf water content of DS and DT after 3 and 5 days without watering. Statistical differences are indicated with lowercase letters determined by Duncan’s test at *p* < 0.05. (**d**) Water loss of DS and DT. All data are shown as means ± SE with three independent biological replicates.

The phenotypes of the candidate lines were further studied and validated at the seedling stage under mild and severe drought conditions. Drought stress intensity was determined by monitoring soil water content (SWC), and mild drought (50~55% SWC) and severe drought (30~35% SWC) were achieved after 3 days and 5 days without watering, in our controlled growth chamber, respectively (Figure 1a). We found that these two lines had no visible phenotypic alterations when exposed to mild drought, though the leaf water content of 88S148 decreased significantly compared with that of the control plants. Under severe drought conditions, no visible phenotypic changes were observed in 14S837, but leaf water content began to decrease. In direct contrast, 88S148 showed severe wilting symptoms and a lower leaf water content (Figure 1b,c). Additionally, the water loss in detached shoots was measured, and the data showed that the shoots of 14S837 lost water more slowly than 88S148 (Figure 1d). With the above data from studying seed and seedling phenotypes under drought stress conditions, 14S837 and 88S148 were selected as DT and DS genotypes of Chinese cabbage, respectively, for subsequent metabolomics studies.

### 2.2. Metabolome Profiling

**Figure 2:**
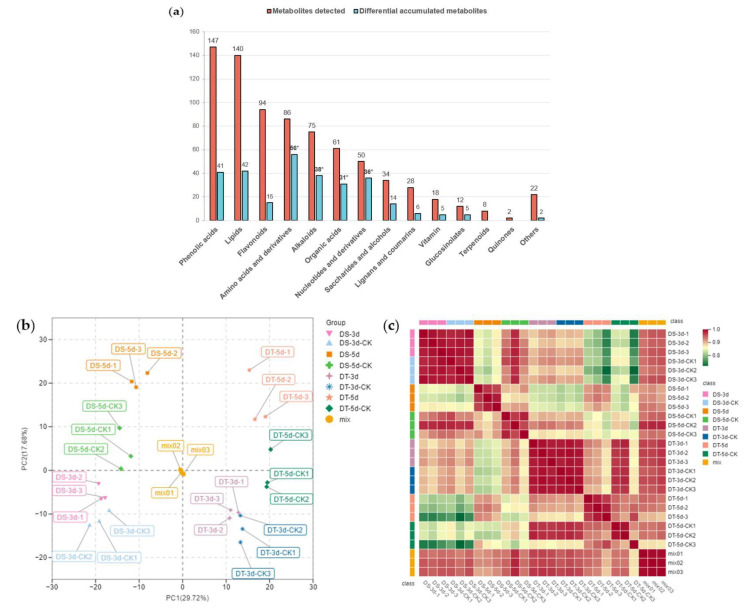
Metabolic profiling of drought−tolerant and susceptible genotypes in response to mild and severe drought stress. (**a**) Number of metabolites and differential metabolites in different classes. The statistical significance was determined via hypergeometric test with * *p* < 0.05; (**b**) Principal component analysis (PCA); (**c**) Heatmap of Pearson’s correlation coefficient. Different colors represent different samples, DS−3d (pink), DS−3d−CK (baby blue), DS−5d(orange), DS−5d−CK (green), DT−3d (purple), DT−3d−CK (dark blue), DT−5d (lightsalmon), DT−5d−CK (dark green), mix (golden yellow).

To investigate the metabolic response to drought stress in Chinese cabbage, leaf samples of DS and DT were collected from day 3 and day 5 drought−treatment plants alongside the controls, and total plant metabolites were extracted and analyzed using a widely targeted metabolome analysis based on an ultra−performance liquid chromatography−tandem mass spectrometry (UPLC−MS/MS) platform. A total of 777 metabolites were detected in all 24 samples in our drought assay, and they could be further divided into 14 classes, including 147 phenolic acids, 140 lipids, 94 flavonoids, 86 amino acids and derivatives, 75 alkaloids, 61 organic acids, 50 nucleotides and derivatives, 34 saccharides and alcohols, 28 lignans and coumarins, 18 vitamins, 12 glucosinolates, eight terpenoids, two quinones, and 22 other metabolites (Figure 2a, Table S1). A total of 371 metabolites were annotated using the Kyoto Encyclopedia of Genes and Genomes (KEGG) compound database, and 252 metabolites were further mapped to plant metabolic pathways using the KEGG pathway database.

The total ion current (TIC) of different quality control (QC) samples showed highly overlapping patterns in retention time and peak intensity, confirming the data was stable and repeatable at the tested time points (Figure S2). Principal component analysis (PCA) was carried out to visualize the overall metabolic differences and relationship among samples. The PCA of all samples (including QC samples) showed little variation within each group, but large variation between groups (Figure 2b). The two major components of PCA explained 47.4% of the total variance, and the first principal component (PC1) explained 29.72% of the total variation, thus separating the two contrasting groups of DS and DT unambiguously. Separation between the drought treatment and control groups indicated drought treatment significantly affected the metabolism of the two lines, suggesting our methodology correctly reveals the metabolic basis of drought stress. A heatmap based on Pearson’s correlation coefficient between all samples (including QC samples) was also constructed (Figure 2c), showing a highly significant positive correlation among three tested biological replicates. On the whole, our experimental design and data are solid and well suited for downstream analysis. 

### 2.3. Differential Metabolites in Multiple Comparison Groups

**Figure 3:**
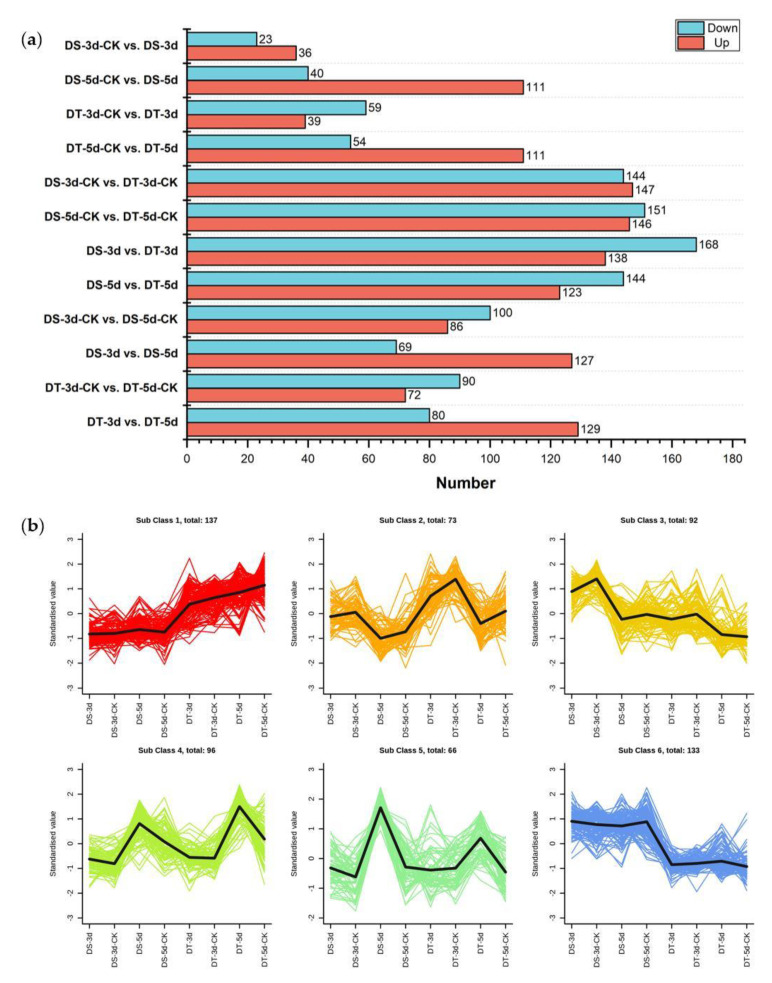
Analysis of differential metabolites (DMs). (**a**) Number of upregulated and downregulated DMs in 12 comparison groups; (**b**) The K−means analysis of DMs.

To identify differential metabolites (DMs), orthogonal partial least squares discriminant analysis (OPLS−DA) was performed in 12 comparison groups and variable importance in projection (VIP) values were obtained. DMs were determined by fold changes (FC) ≥ 1.5 or ≤ 0.67 and VIP ≥ 1, and a total of 597 DMs were identified in all tested comparison groups, providing the core DMs data for subsequent analysis (Figure 3a, Table S2). 

In order to understand the dynamics of DMs in different comparison groups, K−means cluster analysis was performed based on the accumulation patterns of different metabolites, and six sub−classes were identified (Figure 3b, Table S3). A total of 137, 73, 92, 96, 66, and 133 metabolites were clustered from sub−classes 1 to 6, respectively. Notably, DMs in sub−class 4 and 5 show higher accumulation under severe drought conditions, suggesting that these metabolites might be top metabolic candidate biomarkers in the drought response of Chinese cabbage.

### 2.4. Exploration of Key Drought−Responsive Metabolites in Chinese Cabbage

**Figure 4:**
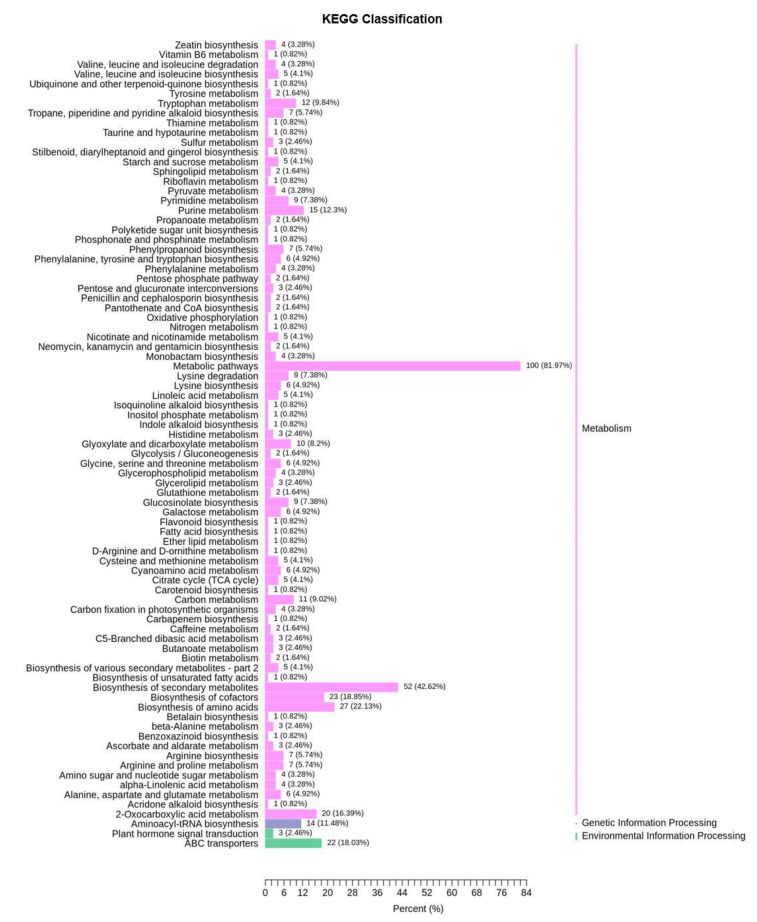
KEGG pathway analysis of 291 differential metabolites of drought−tolerant and susceptible genotypes in response to drought stress.

To explore the metabolic response of Chinese cabbage under drought stress, we first examined the variation of DMs in four comparison groups (DS−3d−CK vs. DS−3d, DS−5d−CK vs. DS−5d, DT−3d−CK vs. DT−3d, DT−5d−CK vs. DT−5d), and a total of 291 DMs were detected in the above four comparison groups (Table S2). Among the 291 metabolites, 27.89% (41/147) phenolic acids, 30% (42/140) lipids, 15.96% (15/94) flavonoids, 65.12% (56/86) amino acids and derivatives, 50.67% (38/75) alkaloids, 50.82% (31/61) organic acids, 72% (36/50) nucleotides and derivatives, 41.18% (14/34) saccharides and alcohols, 21.43% (6/28) lignans and coumarins, 27.78% (5/18) vitamin, 41.67% (5/12) glucosinolates, and 9.09% (2/22) other metabolites, they showed differential accumulation patterns in response to drought stress (Figure 2a). Notably, amino acids and derivatives, alkaloids, organic acids, and nucleotides and derivatives, were significantly affected by drought stress. KEGG pathway enrichment analysis indicated that DMs responding to drought stress were significantly enriched in 13 pathways including purine metabolism, 2−oxocarboxylic acid metabolism, metabolic pathways, glyoxylate and dicarboxylate metabolism, biosynthesis of amino acids, aminoacyl−tRNA biosynthesis, lysine biosynthesis, carbon metabolism, ABC transporters, lysine degradation, the citrate cycle (TCA cycle), arginine biosynthesis, and tryptophan metabolism (Figure 4).

**Figure 5:**
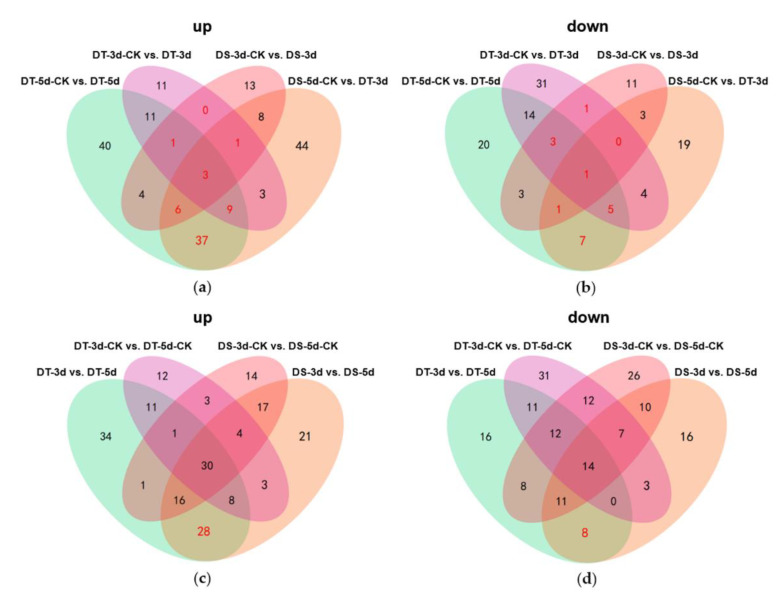
Venn diagrams showing the numbers of upregulated (**a**,**c**) and downregulated (**b**,**d**) differential metabolites (DMs) in different comparison groups. The metabolites labeled in red are considered as the drought−responsive metabolites.

**Figure 6:**
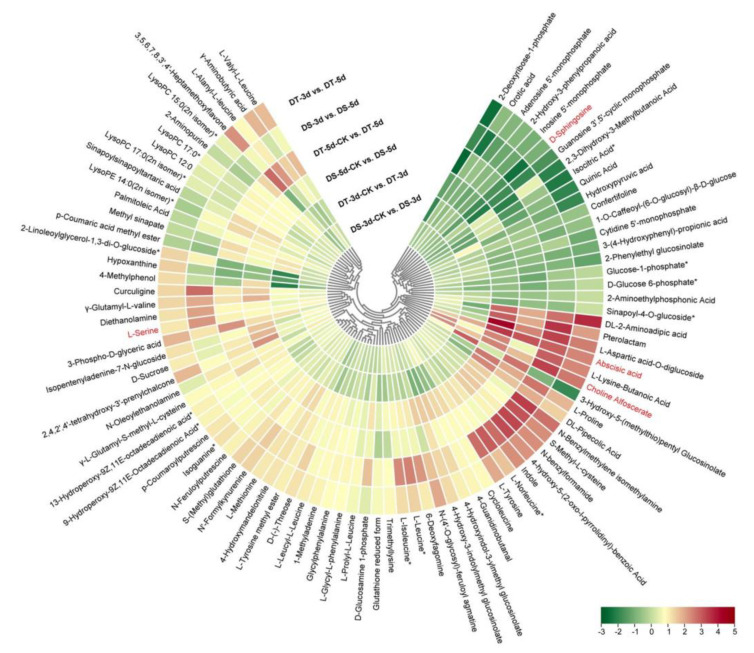
Heatmap of 90 drought−responsive metabolites in Chinese cabbage was constructed based on Log_2_ ratios of fold changes. The metabolites labeled in red were considered as potential biomarkers of drought stress in Chinese cabbage. The Log_2_ (fold changes values) and the color scale are shown at the bottom right of heatmap.

Venn diagrams were constructed to show the number of common DMs in both lines under mild drought and severe drought stress conditions. Five common upregulated and five common downregulated DMs were found in DS and DT under mild drought, while 55 common DMs were upregulated and 14 common DMs were downregulated in both DS and DT under severe drought (Figure 5a,b). Four common DMs in both genotypes and drought conditions, abscisic acid (ABA), serine, choline alfoscerate (GPC), and sphingosine, were proposed as potential biomarkers of drought stress in Chinese cabbage. Furthermore, 28 common upregulated and eight common downregulated DMs were specifically altered in DS−3d vs. DS−5d and DT−3d vs. DT−5d but not in DS−3d−CK vs. DS−5d−CK and DT−3d−CK vs. DT−5d−CK (Figure 5c,d, Table S2). These common DMs with similar response patterns to drought stress in both genotypes were considered to be the drought−responsive metabolites. By removing the duplicated metabolites, a total of 90 metabolites, with 68 upregulated and 22 downregulated ones, were selected and proposed as the drought−responsive metabolites in Chinese cabbage (Figure 6, Table S4). Among these drought−responsive metabolites, 24 metabolites were classified as amino acids and derivatives. Thus, we further investigated the levels of free amino acids under drought stress. Serine levels were increased in both drought treatments and genotypes. Proline, leucine, isoleucine, methionine, and tyrosine showed a similar response pattern with higher accumulation under severe drought treatment in both genotypes. The aspartic acid levels were higher in DT irrelevant of drought treatment and were specifically decreased in DT upon severe drought. Other amino acids, including threonine, asparagine, cysteine and glutamic acid, were found to be unaffected by drought stress. 

In conclusion, we identified a large number of metabolites involved in the drought responses of Chinese cabbage, among which, amino acids and derivatives played more important roles in the drought responses.

### 2.5. Differential Drought Responses between DS and DT Genotypes

**Figure 7:**
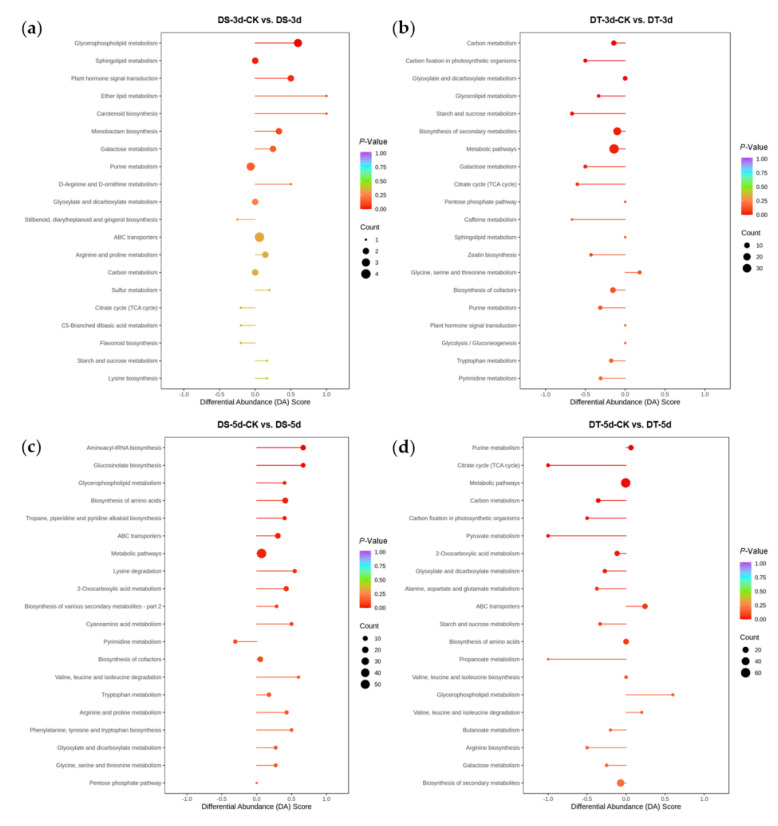
KEGG pathway enrichment analysis and differential abundance (DA) scores of differential metabolites (DMs) in four comparison groups. (**a**) DS−3d−CK vs. DS−3d; (**b**) DT−3d−CK vs. DT−3d; (**c**) DS−5d−CK vs. DS−5d; (**d**) DT−5d−CK vs. DT−5d.

In order to study the differential response of DS and DT to drought stress, KEGG pathway enrichment analysis was performed for DMs in four comparison groups (Figure 7). 59 DMs (36 upregulated and 23 downregulated) were found in DS−3d−CK vs. DS−3d, which were enriched in glycerophospholipid metabolism, sphingolipid metabolism, and plant hormone signal transduction, whereas 98 DMs (39 upregulated and 59 downregulated) were found in DT−3d−CK vs. DT−3d enriched in carbon metabolism, carbon fixation in photosynthetic organisms, glyoxylate and dicarboxylate metabolism, glycerolipid metabolism, starch and sucrose metabolism, biosynthesis of secondary metabolites, metabolic pathways, galactose metabolism, and the TCA cycle (Figure 3a and Figure 7a,b). In the mild drought stress, there were more DMs in DT, especially more downregulated DMs, indicating that mild drought stress had a stronger effect on DT. 

Under 5−day drought treatment, there were 151 DMs (111 upregulated and 40 downregulated) in DS, which mainly involved in aminoacyl−tRNA biosynthesis, glucosinolate biosynthesis, glycerophospholipid metabolism, biosynthesis of amino acids, tropane, piperidine, pyridine alkaloid biosynthesis, ABC transporters, metabolic pathways, lysine degradation, and 2−oxocarboxylic acid metabolism. In comparison, 165 DMs (111 upregulated and 54 downregulated) were identified in DT, enriched in purine metabolism, TCA cycle, metabolic pathways, carbon metabolism, pyruvate metabolism, carbon fixation in photosynthetic organisms, 2−oxocarboxylic acid metabolism, and glyoxylate and dicarboxylate metabolism (Figure 3a and Figure 7c,d). Notably, the differential abundance (DA) scores of DT were mostly negative in the enrichment pathways under mild drought and severe drought conditions (Figure 7b,d), while the scores in DS were mostly positive in the enrichment pathways (Figure 7a,c).

**Figure 8:**
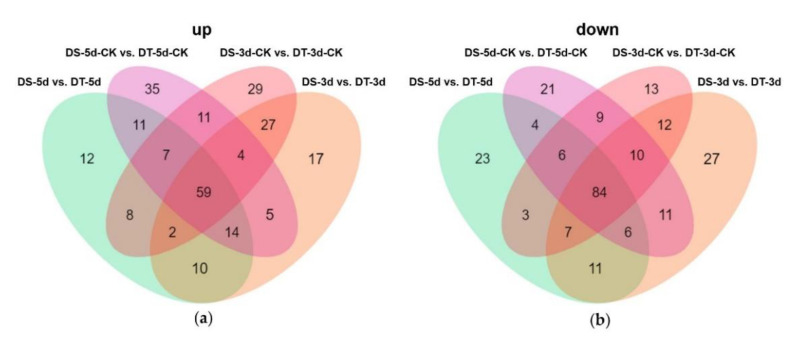
Venn diagram showing the number of upregulated (**a**) and downregulated (**b**) differential metabolites (DMs) between DS and DT under control and drought conditions.

To further explore the different metabolic response patterns of the two selected contrasting Chinese cabbage genotypes, a comparative metabolic analysis between DS and DT was performed (Figure 8, Table S2). Fifty−nine metabolites accumulated more in DT, especially some of the key drought−responsive metabolites, such as ABA, GPC, and reduced glutathione, which may explain the increased drought tolerance observed in DT (Figure 8a). Furthermore, 84 common DMs showed higher accumulation in DS in both drought and control conditions compared with the ones in DT, including 25 phenolic acids and 25 flavonoids, thus indicating DS contains more phenolic acids and flavonoids (Figure 8b).

## 3. Discussion

As sessile organisms, plants need to respond to the great challenges of environmental stresses, among which, drought is one of the most severe ones, especially in the large arid and semi−arid areas located primarily in the developing countries and regions of the world [2]. Understanding the plants’ drought responses can not only help our understanding of how different plant species mitigate drought stresses, but also provide insights to crop breeding for high water use efficiency and for crop security within economically vulnerable populations. Metabolites are the key tools for plants to facilitate water use, especially in leafy vegetables like Chinese cabbage, which require large quantities of water but have a limited ability to extract water from soil. In this current study, we analyzed the metabolome of two Chinese cabbage genotypes with different drought tolerance abilities and found that amino acids and derivatives, alkaloids, organic acids, and nucleotides and derivatives in Chinese cabbage were significantly affected by drought stress. A total of 777 metabolites were detected by a widely targeted metabolome and 90 metabolites were selected and proposed as the drought−responsive metabolites in Chinese cabbage, with ABA, serine, choline alfoscerate, and sphingosine as potential representative drought stress biomarkers. In addition, we also found that DS and DT have different metabolite response patterns and contrasting coping strategies in response to drought stress, which might explain their corresponding phenotypic differences under drought stress. Notably, constitutively high levels of ABA and glutathione were detected in the drought−tolerant genotype in all tested and control conditions, which might help explain the increased drought tolerance of DT. A simplified drought response model in Chinese cabbage was proposed according to our study.

The plant hormone ABA plays a central role in response to abiotic stress by modulating stomata, plant growth, and metabolic pathways [15,16]. In our study, ABA was shown to be the most important metabolite in Chinese cabbage for responding to drought stress. We found that the level of ABA increased rapidly under drought stress in both genotypes. More importantly, the level of ABA in DT was higher than the one in DS under both drought and control conditions. Thus, the differential levels of ABA may explain the contrasting phenotypic differences observed, though the mechanism of ABA accumulation in DT requires further investigation.

Some important intermediates in the TCA cycle, including citric acid, isocitric acid, α−ketoglutaric acid, succinic acid, and malic acid, were shown to have a lower accumulation in DT under drought stress, indicating that the TCA cycle was inhibited by water deficiency. This is consistent with downregulation of enriched metabolic pathways in DT and may be related to the drought acclimation ability of DT. Furthermore, it has been shown that citric acid can confer abiotic stress tolerance in plants, and exogenous citric acid application can also enhance drought tolerance in a variety of plant species [17,18,19,20]. Therefore, increasing endogenous citric acid level by exogenous spray may be a potentially useful approach for improving the drought tolerance of Chinese cabbage.

Proline has been reported in multiple studies as another important drought−responsive metabolite [13,21,22,23]. Beyond acting as an osmolyte for osmotic adjustment, proline also stabilizes sub−cellular structures and contributes to reactive oxygen species (ROS) detoxification [24]. Although we did not observe changes in proline levels under mild drought stress, the concentration increased under severe drought condition in both genotypes. Serine, which is required for cysteine and methionine biosynthesis, was proposed as one of the potential biomarkers of drought stress in Chinese cabbage. Methionine, the precursor of aliphatic glucosinolates, showed increased levels under severe drought condition in both DS and DT, which may explain why the aliphatic glucosinolates increased under drought stress. Aspartic acid can be transformed from oxaloacetate, an intermediate in the TCA cycle that can be catalyzed by aspartate aminotransferase [25]. Thus, the decreased levels of aspartic acid in DT may be a result of downregulation of other intermediates in the TCA cycle.

Glutathione (GSH) is a non−protein tripeptide involved in the detoxification of excess ROS, maintaining cellular redox homeostasis and regulating protein function in plants under abiotic and biotic stresses [26,27,28]. Reduced GSH is oxidized to disulfide (GSSG) during ROS scavenging, and GSSG is recycled to GSH by glutathione reductase [24]. Previous studies have shown that the levels of GSH increase in response to drought stress [29]. Furthermore, exogenously applied and endogenously increased GSH can improve drought stress tolerance in many plant species [27,30,31,32]. While we found no significant changes in the content of GSH in our drought assay, the plants accumulated more GSH in the severe drought stress condition. Moreover, the level of GSH was higher in DT independent of the stress, which may be related to its increased drought tolerance. Furthermore, S−(methyl) glutathione, the thioether of glutathione [33], was induced by drought stress, and its role in the drought response of Chinese cabbage warrants further investigation.

Soluble sugars mainly include sucrose and its products glucose and fructose [34]. It was found that drought increased the amount of soluble sugar in Chinese cabbage [35]. In this study, we discovered that drought stress increased sucrose levels in Chinese cabbage but did not significantly affect the levels of glucose and fructose, which suggests that sucrose plays important roles in osmotic adaption under drought stress.

Phenolic acids, a major class of polyphenols, are constitutively present in vegetables with strong antioxidant activity [36,37]. Drought stress enhanced the accumulation of phenolic acids [29,38]. In this study, we identified a total of 147 phenolic acids, and nine phenolic acids were considered to be involved in the response to drought stress of Chinese cabbage, including 4−methylphenol, 2−hydroxy−3−phenylpropanoic acid, 3−(4−hydroxyphenyl)−propionic acid, p−coumaric acid methyl ester, methyl sinapate, sinapoyl−4−O−glucoside, curculigine, sinapoylsinapoyltartaric acid, and 1−O−caffeoyl−(6−O−glucosyl)−β−D−glucose. Interestingly, we found that nearly half of the 39 upregulated metabolites in the DT−3d−CK vs. DT−3d comparison group were phenolic acids, which may improve the drought tolerance of DT by increasing antioxidant activity in the early stages of drought stress (Table S2).

Pipecolic acid is a lysine−derived non−protein amino acid which regulates plant systemic acquired resistance and basal immunity to bacterial pathogen infection [39]. Recent study has shown that pipecolic acid plays a negative regulatory role in drought tolerance of tomato plants [40]. In this study, the level of DL−pipecolic acid in DT decreased under mild drought stress, which may be related to the drought resistance response in DT. 

Quinic acid is a cyclic carboxylic acid involved in the shikimate pathway [41]. It was reported that quinic acid is the main contributor to the osmotic potential of Quercus suber leaves [42], and its concentration increased under drought stress in some species [23,43]. However, the level of quinic acid decreased in both genotypes under mild drought and further decreased with the aggravation of drought in DT. This result was in accordance with a report on broccoli [22], Salvia miltiorrhiza Bunge (Danshen) [44], peach [45] and maize [46]. Especially in broccoli, low levels of quinic acid were considered to be an important signature of drought tolerance [22]. Consequently, the effect of quinic acid in response to drought needs to be further studied.

γ−aminobutyric acid (GABA), a non−protein amino acid, functions as an intrinsic signaling molecule and accumulates quickly in response to a variety of abiotic stresses in plants [47,48,49]. Research has suggested that GABA can reduce stomatal opening and transpiration water loss by negatively regulating the activity of a stomatal guard cell tonoplast−localized anion transporter ALMT9, thus improving water use efficiency and drought tolerance [50]. Exogenous GABA application can increase drought tolerance in many plant species, e.g., white clover [51], creeping bentgrass [52], and snap bean [53]. However, little is known about the roles of GABA in Chinese cabbage. To our knowledge, this study is the first to report that drought stress can induce GABA accumulation in Chinese cabbage. In parallel with previous research, the roles and mechanisms of GABA in the drought response of Chinese cabbage must be further studied.

**Figure 9:**
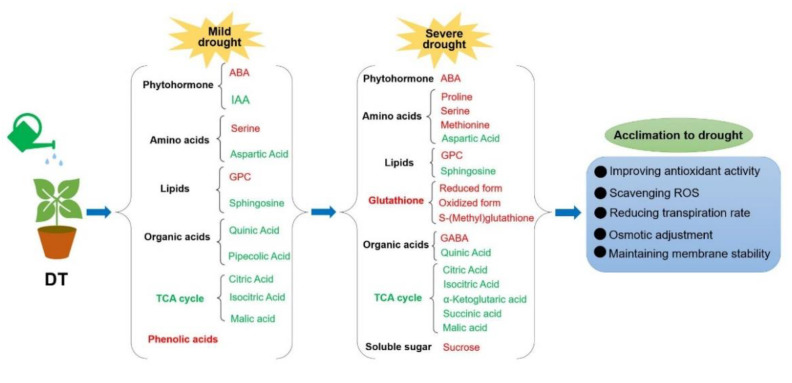
The outline of drought−tolerant genotype responses to drought stress. The red represents increased, while the green represents decreased. ABA, abscisic acid; IAA, indole 3−acetic acid; GPC, choline alfoscerate; TCA cycle, citrate cycle; GABA, γ−aminobutyric acid; ROS, reactive oxygen species.

Based on our findings, we propose a simplified model on how the drought−tolerant genotype of Chinese cabbage responds to mild and severe stresses at the metabolic level (Figure 9).

## 4. Materials and Methods

### 4.1. Plant Materials and PEG Treatment

The seeds of 27 Chinese cabbage inbred lines, including 88S148 and 14S837, were provided by the Chinese cabbage research group, College of Horticulture, Northwest A&F University, Yangling, China.

The drought assay was conducted by screening seed germination under 20% PEG6000−induced osmotic stress. In brief, 30 seeds of the same size, that were full and disease−free were picked and disinfected with 75% ethanol for 1 min, followed by 10% sodium hypochlorite solution (NaClO) for 15 min, then rinsed repeatedly with sterilized water. Next, the disinfected seeds were spread in a 9 cm diameter petri dish containing three filter papers as the germination bed, and soaked with 10 mL 20% PEG6000 (10 mL sterile water as the control), and germinated in a 25 °C light incubator (Dongnan Instrument Co., Ltd. Ningbo, China). A 1 mL PEG solution per day (sterile water for control) was supplemented to each dish during germination. The germination rate was counted on the seventh day and three independent biological replicates were performed. All reagents are purchased from Sangon Biotech Co., Ltd. (Shanghai, China).

### 4.2. Growth Conditions and Drought Treatment

The seeds were sown in 7 cm pots containing a soil matrix of equal weight. Seedlings were grown in the light incubator (Dongnan Instrument Co., Ltd. Ningbo, China) at 25 °C with 14 h light (150 μmol m^−2^s^−1^ of light intensity)/10 h dark cycles and watered normally.

Drought treatment was simulated by a water deficit. Briefly, the seedlings were grown under the conditions described above. The 3−week−old seedlings were watered until the soil was saturated and did not contain much water. Subsequently, the seedlings undergoing drought treatment were no longer watered after removing excess water from the tray.

### 4.3. Water Loss, Soil Water Content and Leaf Water Content Measurement

Water loss was measured according to a previous method [54]. Briefly, the detached shoots of 3−week−old seedlings were immediately weighed and recorded eight times at one−hour intervals. Water loss was expressed as the percentage of initial fresh weight. Three replicates were performed and each replicate contained six individual plants.

Soil water content was determined by the oven drying method. In brief, the fresh weight of the soil matrix in the pot was weighed after removing the roots, then the soil was oven dried at 105 °C for 12 h and weighed again. The soil water content was expressed by the percentage of weight lost out of the initial fresh weight and recorded at 24 h intervals. Three replicates were performed and each replicate contained three individual samples.

For the measurement of leaf water content, the second leaves were collected and immediately weighed to obtain the fresh weight (FW), then the leaves were oven dried at 80 °C for 24 h and dry weights (DW) were measured. Leaf water content (%) was calculated as [(FW − DW)/FW] × 100. Three replicates were performed and each replicate contained three individual plants.

### 4.4. Sample Collection

The seedlings of drought−tolerant and susceptible genotypes were grown in a light incubator with the conditions described above. The second intact leaves counted from outside to inside were collected from five individual plants when the soil water content reached 50~55% (drought for 3 d) and 30~35% (drought for 5 d), respectively. The plants with normal irrigation were also sampled as controls (CK) at the same time. Therefore, a total of four group samples were collected, designated as DS−3d/DS−3d−CK, DS−5d/DS−5d−CK, DT−3d/DT−3d−CK, DT−5d/DT−5d−CK, and three independent biological replicates were performed. The collected leaves were snap−frozen in liquid nitrogen immediately and stored at −80 °C until extraction. 

### 4.5. Metabolome Profiling

Widely targeted metabolome was employed to acquire the metabolomic profile of samples conducted by Wuhan Metware Biotechnology Co., Ltd. (Wuhan, China). In brief, the sample leaves were freeze−dried by a vacuum freeze−dryer (Scientz−100F, Ningbo, China) and crushed using a mixer mill (MM 400, Retsch, Germany) at 30 Hz for 90 s. Lyophilized powder (100 mg) was weighed and dissolved with 1.2 mL 70% methanol solution (vortex 30 s every 30 min and repeated six times), followed by overnight extraction at 4 °C. Finally, the extract was centrifuged at 12,000 rpm for 10 min, and the supernatant was collected and filtered through a filter membrane (0.22 μm pore size, ANPEL, Shanghai, China) before UPLC−MS/MS analysis.

The sample extracts were analyzed using an UPLC−ESI−MS/MS system (UPLC, SHIMADZU Nexera X2, Kyoto, Japan; MS, Applied Biosystems 4500 Q TRAP, Waltham, MA, USA). Quality control samples (QC) prepared by mixing all sample extracts were inserted every 10 samples to monitor the repeatability of the analysis process. UPLC separation was performed with the same protocol as Yuan et al. [55]. Mass spectrographic analysis was performed by a triple quadrupole–linear ion trap mass spectrometer equipped with an ESI Turbo Ion−Spray interface (Applied Biosystems 4500 Q TRAP UPLC/MS/MS System), which operated according to a previous study [56]. The mass spectrometry data was processed with the Analyst 1.6.3 software (AB Sciex). Qualitative analysis of metabolites was identified based on MetWare database (Wuhan Metware Biotechnology Co., Ltd., Wuhan, China) and other public databases. Quantification of metabolites was carried out using multiple reaction monitoring method [56].

### 4.6. Data Analysis

Identified metabolites were annotated using the KEGG compound database (http://www.kegg.jp/kegg/compound/, accessed on 15 November 2021); annotated metabolites were then mapped to the KEGG pathway database (http://www.kegg.jp/kegg/pathway.html, accessed on 15 November 2021) [57]. KEGG pathway enrichment analysis was performed using the Metware Cloud, a free online platform for data analysis (https://cloud.metware.cn, accessed on 10 January 2022) and significance was determined by hypergeometric test. The differential abundance (DA) score is a metabolic change analysis method based on a pathway which can reflect the overall changes of all differential metabolites in a pathway. DA score is calculated as (number of upregulated DMs annotated to a pathway−number of downregulated DMs annotated to the pathway)/number of all metabolites annotated to the pathway.

Multivariate statistical analysis can simplify the high−dimensional and complex data, and retain the original information to the greatest extent. PCA, a classic unsupervised pattern recognition multivariate statistical analysis method, was performed by the prcomp function within R (www.r-project.org, accessed on 15 November 2021). The data was unit variance scaled before PCA. OPLS−DA, a supervised pattern recognition method, was performed by R software using R package MetaboAnalystR [58]. The data was log transformed (log2) and mean centered before OPLS−DA. In order to avoid overfitting, a permutation test (200 permutations) was performed. VIP values were extracted from the OPLS−DA result to identify DMs.

Pearson correlation coefficients (PCC) and K−means cluster analysis were also performed with the Metware Cloud. For the K−Means analysis, the relative contents of DMs in all groups were standardized using the Z−score algorithm. A heatmap of metabolites was displayed by TBtools [59].

## Data Availability

Not applicable.

## Supplementary Materials

The following supporting information can be downloaded at: https://www.mdpi.com/article/10.3390/ijms23115947/s1.
