# Supplementary figures and images for: Comparative Metabolic Study of Two Contrasting Chinese Cabbage Genotypes under Mild and Severe Drought Stress
Source: Int J Mol Sci. 2022 May 25;23(11):5947. doi: 10.3390/ijms23115947 (PMC9180449; doi:10.3390/ijms23115947)

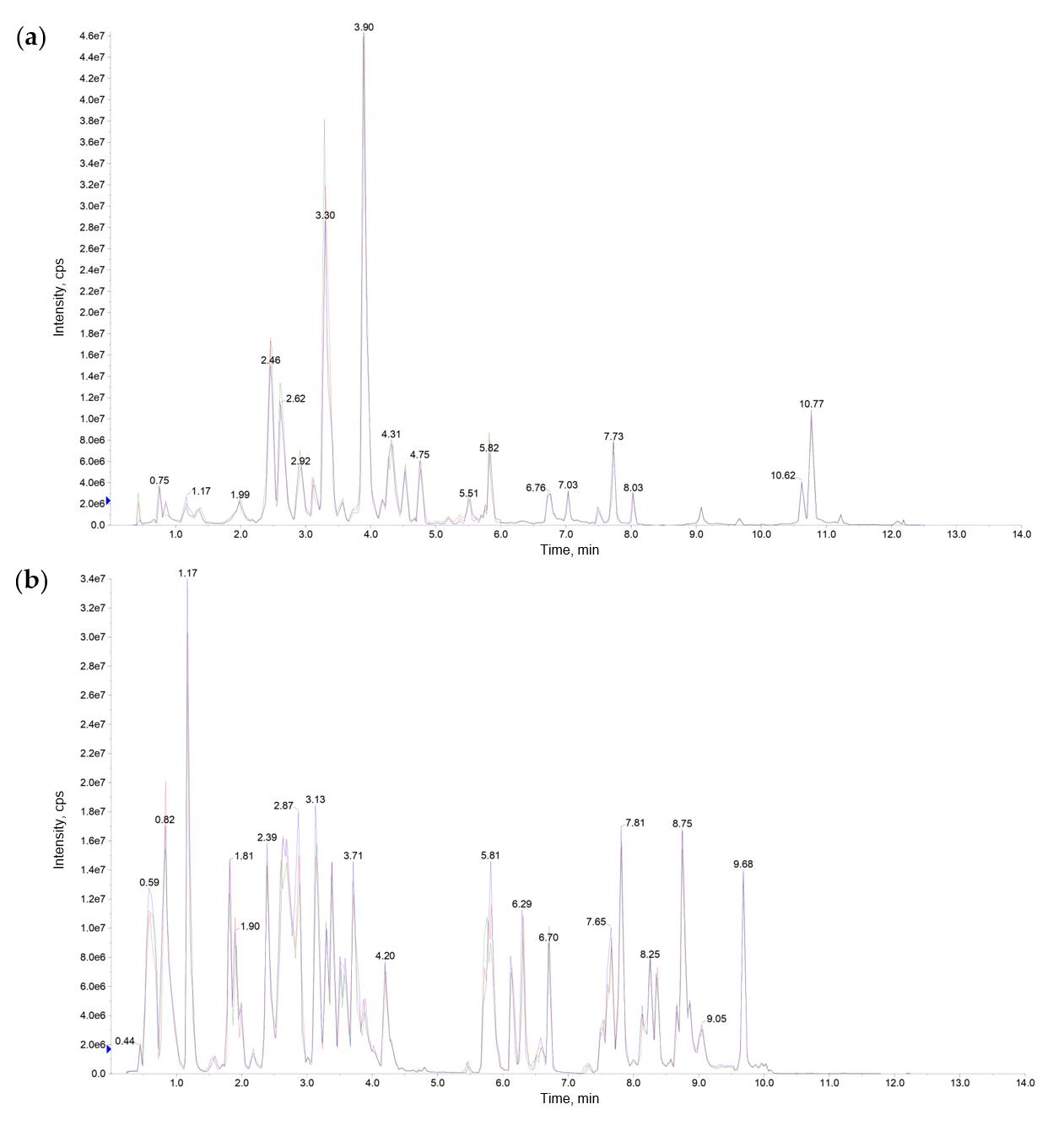

Supplement: Supplementary file 1 [file ijms-23-05947-s001.zip › Figure S2.jpg]

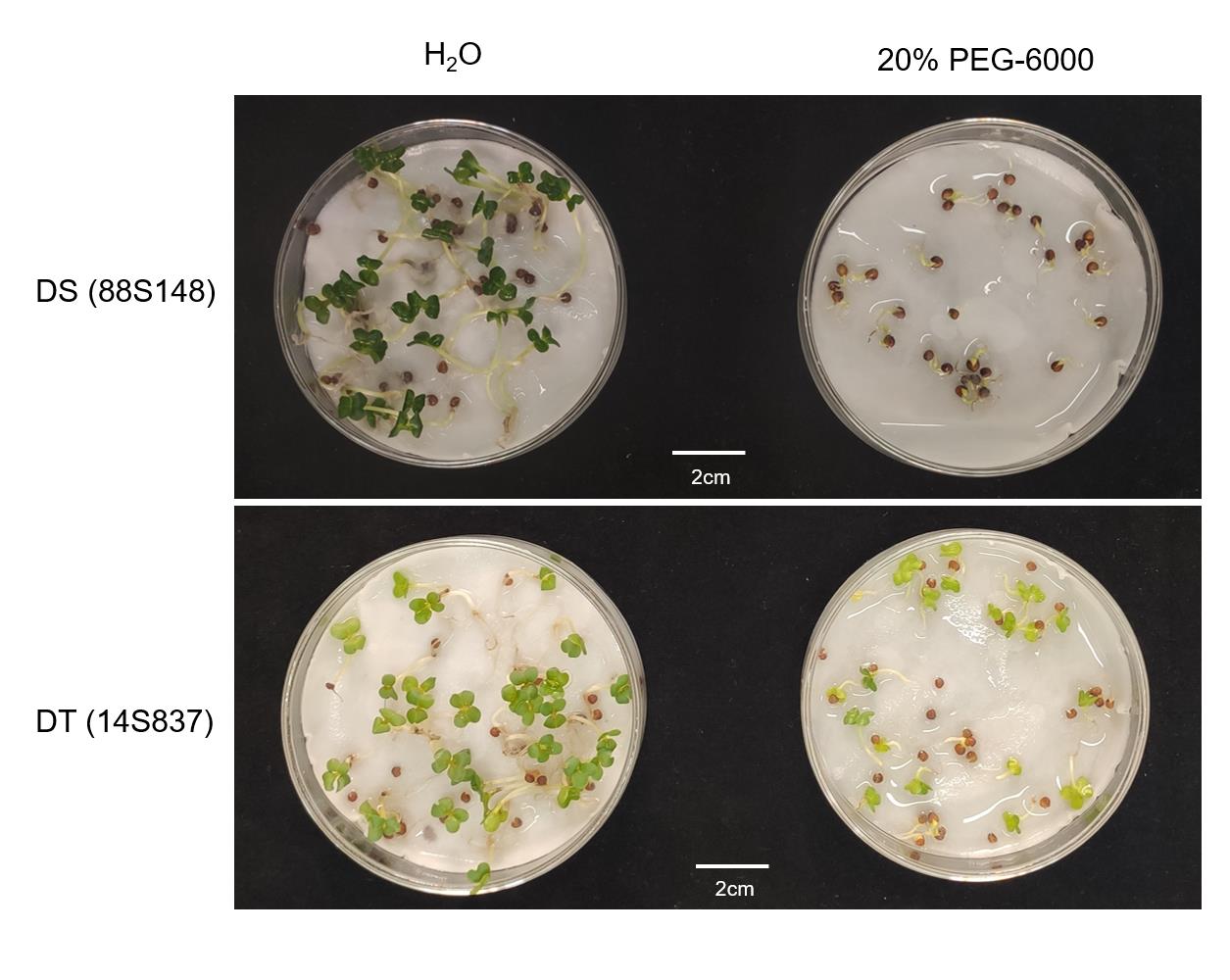

Supplement: Supplementary file 1 [file ijms-23-05947-s001.zip › Figure S1.jpg]
